# Supplementary material for: Greb1 is required for axial elongation and segmentation in vertebrate embryos
Source: Biol Open. 2020 Feb 11;9(2):bio047290. doi: 10.1242/bio.047290 (PMC7044451; doi:10.1242/bio.047290)
Supplement: Supplementary information [file biolopen-9-047290-s1.pdf]

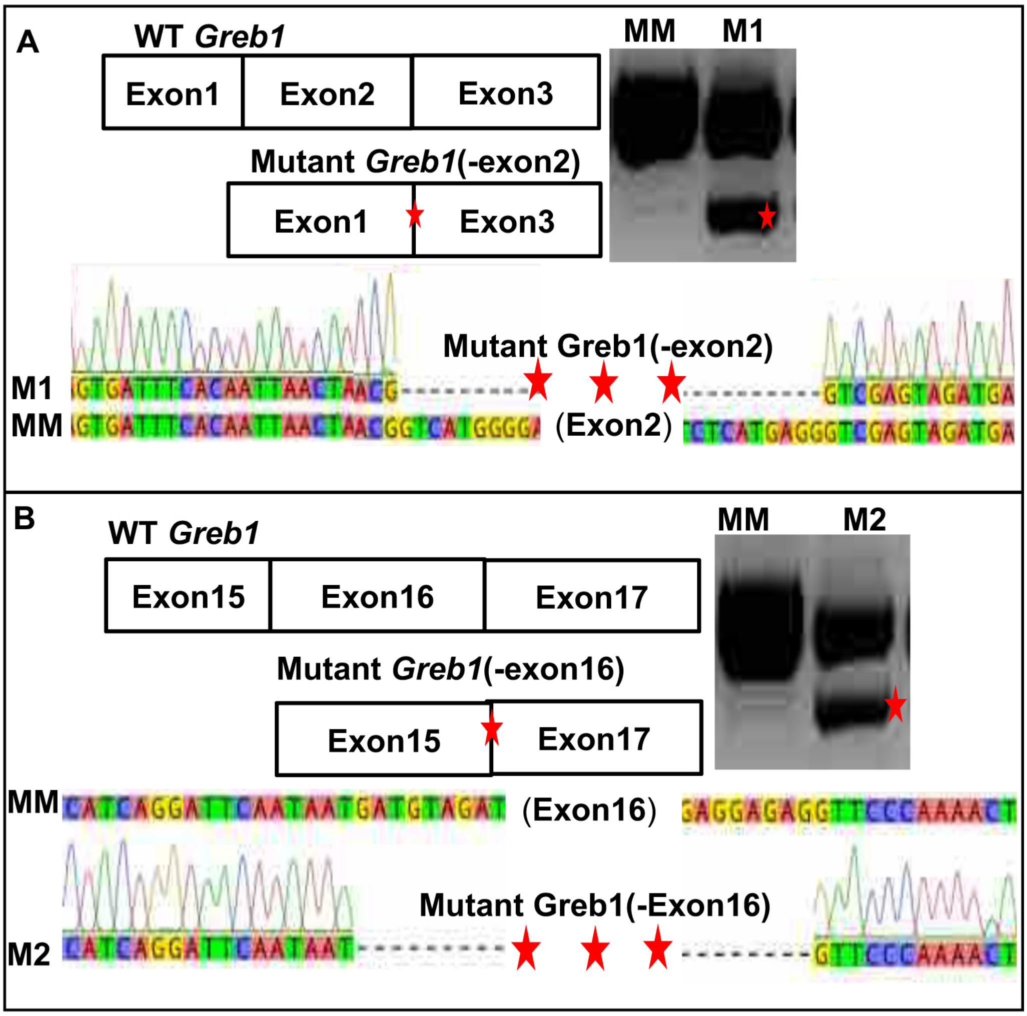

Figure S1

Fig. S1: M1 and M2 morpholinos specifically knockdown *Greb1*: (A) is schematics of *greb1* exon1-3, exon 1 and 3; and 1% agarose gel that shows wildtype and mis-spliced (red star) RT-PCR products. M1 morpholino targets exon2-intron2 boundary, and MM is control morpholino (5-nucleotide mis-matched of M2) chromatogram was obtained from Sanger sequencing for mis-spliced product-deleted exon2. B same as A and B but for M2 morpholino that targets exon16-intron16 boundary.

Biology Open • Supplementary information

Table S1: A list of differentially expressed genes. Tab1 pairwise comparisons of CNH vs PSM, and CNH vs TMB. Tab2 differentially expressed gene in CNH vs TBM comparison.

Click here to Download Table S1

Table S2: The mouse genome informatics (MGI) data show expression of differentially expressed genes in various tissues. Tab1 list of differentially expressed genes and the annotated tissues where these genes express.Green colour show CNH up regulated and red colour CNH down regulated genes. Tab2 and tab3 graphical representation differentially expression genes in various tissues, it is an output from MGI.

Click here to Download Table S2

Table S3: A comparison of CNH enriched genes with published CNH/NMP transcriptome.Tab1 comparison among CNH and published CNH and NMP data. Tab2 notochord genes which are also enriched in CNH.

CNH-10.5 (Prajapati et al)	Notochrod genes(Wymeer	"CNH-10.5" and "Notochor	Neural-Tube (PNT Enriched	"CNH-10.5" and "PNT"	SZ Genes (Pnt Enriched Genes Olivera-Martinez Et	"CNH-10.5" and "SZ":
Defcr26	Defcr26	Defcr26	Abat	Olfml3	Adamts6	Cdx2
Defcr-rs7	Defcr-rs7	Defcr-rs7	Ace2		Aggf1	Cyp26a1
Hap1	Hap1	Hap1	Adcyap1r1		Akap12	Evx1
Hbb-b1	Hbb-b1	Hbb-b1	Alcam		Ankrd50	Fgf8
Klhl6	Klhl6	Klhl6	Ankh		Aplp2	Fzd10
Ndufaf4	Ndufaf4	Ndufaf4	Apoh		Apod	Gja1
Prnp	Prnp	Prnp	Aqp1		Arhgap8	Greb1
Scara3	Scara3	Scara3	Arhgap17		Arid1b	Hoxc9
Shh	Shh	Shh	Arl4c		Arsb	Il17rd
Slit2	Slit2	Slit2	Asl1		Astl	Rspo3
A830080H07Rik	4930533K18Rik		Atl1		Axin2	Slc1a4
Acot1	Arl4a		C13orf15		Bambi	T
Acot7	Bicc1		C1h12orf23		Bbx	Wnt3a
Adamtsl2	Btg1		C1orf53		Bmp2	Wnt5b
Adrb2	Chrd		C3h2orf43		Bms1	
Angpt2	Clusterin		C7orf23		Brwd1	
Anxa3	Cobl		C8orf4		C1orf69	
Apcdd1	Col8a2		C9orf19		C9orf89	
AY761185	Cthrc1		C9orf30		Caprin2	
Bex4	Emp1		Ca2		Cbln1	
Bik	Fermt1		Cap2		Ccdc28a	
C230098O21Rik	Fltr3		Ccdc93		Ccdc82	
Car14	Foxa1		Cdh4		Ccdc88a	
Ccnd1	Igfbp5		Cited4		Cd3e	
Cd40	Lmx1a		Ckb		Cdx2	
Cd63	Ly-6H		Cnot6		Clic4	
Cd83	Moxd1		Coch		Cmtm8	
Cdk6	Nav3		Col14a1		Cnot2	
Cdkn1a	Noto		Ctdspl		Comtd1	
Cdkn1c	Nrep		Dbc1		Crhbp	
Cdx2	Prickle2		Dhrs3		Csrnp1	
Centd3	Ptch1		Dio2		Cwc27	
Chic1	Rprm		Ednra		Cyp26a1	
Chst7	scl0003799.1_2		Efemp1		Cyp27c1	
Cib1	Sema5a		Eml1		Cytl1	
Cpn1	Smoc1		Eml4		Dach1	
Creld1	Sox9		Fabp5		Dbf4	
Crip2	Spef1		Fam5b		Dcbld1	
Cyp26a1	Synpo		Fgd4		Dll1	
D17Wsu92e	Timp3		Fgfr2		Dock7	
Ddah1			Flt4		Edaradd	
Defcr3			Fn1		Egr1	
Defcr6			Fzd7		Enc1	
Defcr-rs10			Gcnt7		Epha1	
Defcr-rs2			Gdpd4		Epha4	
Dock6			Gopc		Esrrg	
Dusp4			Grm4		Etv1	
Dusp6			Gtf2h4		Evx1	
Elf3			Gucy1a3		F2rl1	
Endod1			Ifnar2		Fabp6	
Eno3			Itga8		Fam171b	
Ets2			Kcnab1		Farp1	
Etsrp71			Krt14		Fgf1	
Etv4			Lgi1		Fgf18	
Eva1b			Lims1		Fgf3	
Evx1			Lmo4		Fgf8	
Fgf17			Lmx1b		Fkbp7	
Fgf8			Loc395991		Frmd4b	
Fnbp1			Loc396300		Fzd10	
Foxa3			Loc416235		Gas2	
Furin			Loc421845		Gfra1	
Fzd10			Loc422305		Gja1	
Gabarapl1			Loc769266		Gna11	
Gad1			Loc769421		Gnai3	
Gcnt2			Lrrn1		Gnot1	
Gja1			Ltbp1		Greb1	
Gldc			Ly6e		Hapln1	
Gpr83			Manea		Has2	
Greb1			Mapkap1		Hoxc9	
Grina			Metrnl		Hsf5	
Hba-x			Muc5b		Il17rd	
Hopx			Mylk		Il1r1	
Hoxa11s			Myo3a		Itga4	
Hoxa7			Ndnf		Kat6a	
Hoxb7			Neurog1		Kiaa0226l	
Hoxc10			Nkx6-2		Lef1	
Hoxc6			Nlgn1		Lhfpl2	
Hoxc9			Npr3		Loc427799	
Hoxd10			Nr6a1		Lrig3	
Hoxd12			Olfm1		Lrrc42	
Iap			Olfml3		Lrrc45	
Il17rd			Pbx3		Map3k5	
Insr			Pdgfb		March6	
Irf1			Pla1a		Mettl6	
Kdm7a			Plek2		Mid1ip1	
Lhpp			Plscr4		Mllt3	
Limch1			Ppap2a		Mpzl2	
Lix1			Prdm2		Msgn1	
Lmo2			Pros1		Msx1	
LOC100044177			Prtg		Msx2	
LOC100044289			Ptn		Mtif2	
LOC100045413			Pts		Myc	
LOC100045869			Rarb		Myof	
LOC100046775			Rbm24		Nefm	
LOC212390			Rcn1		Nell2	
Ly6a			Rffl		Nipa1	
Lypd6b			Rfx4		Nop14	
Mgst1			Robo2		Nsg1	
Mmp2			Ror1		Nsun2	
Mylc2b			Sdc2		Nsun3	
Ngfr			Sdpr		Ntm	
Notch4			Sfrp2		Pcyt1b	
Notum			Shroom1		Pdap1	
Nup210			Slc39a8		Pdcd2	
Oat			Slc41a2		Pde10a	
Ogfrl1			Slc7a9		Pitpnc1	
Olfml3			Smad6		Rai14	
Pdia5			Snai2		Rasgrp3	
Pgk1			Sntb1		Rbm35b	
Pgm2			Sox18		Rgs20	
Pitrm1			Sox3		Rock2	
Prr18			Spag9		Rora	
Rab8b			Sptlc3		Rrbp1	
Ramp2			Sqrdl		Rspo3	
Rasgrp4			St5		S100z	
Rell1			St6galnac2		Scml2	
Rnf208			Ston2		Sdc3	
Rps6ka1			Tiam2		Senp5	
Rspo3			Timp3		Slc1a4	
Sall3			Trib2		Slc37a3	
Samd9l			Ubxd4		Snai1	
Sbk			Wfdc1		Spry2	
Scara5			Zeb2		Srd5a3	
Sct					Suclg2	
Serpine2					Syndig1	
Sgk1					Sypl1	
Shc1					Syt10	
Slc1a3					T	
Slc1a4					Thbs4	
Slc2a3					Tmem100	
Smox					Tmem45a	
Snx11					Tnpo1	
Sp5					Tuba4a	
Strbp					Unc5b	
Susd4					Upp1	
T					Urb2	
Tcea3					Vav3	
Thsd2					Wdr66	
Tmprss2					Wnk2	
Tpd52					Wnt3a	
Tpm1					Wnt5a	
Tril					Wnt5b	
Trim2					Xpc	
Uap1l1					Ythdc1	
Wfdc2					Zeb1	
Wnt3a						
Wnt5b						
Zcchc18						
Zyx						

Table S4: Results of pathway enrichment analysis (Supplementary Methods) showing the top ten enriched pathways and their associated genes.

P-2, WNT, Cyclin D1

Enrichment analysis report

Enrichment by Pathway Maps

#	Maps	Total	min(pValue)	Min FDR	(1) CNH Vs PSM.diffGenes.up p-value	FDR	In Data	Genes from Active Data	(2) CNH Vs PSM.diffGenes.dn p-value	FDR	In Data	Genes from Active Data
1	Development_Notch Signaling Pathway	43	7.829E-09	8.3766E-07	2.594E-01	3.707E-01	1	Furin	7.829E-09	8.377E-07	6	p63, NOTCH1 (NICD), SAP30, NOTCH1 (NEXT), NOTCH1 precursor, NOTCH1 receptor
2	Transcription_Androgen Receptor nuclear signaling	45	5.702E-07	0.00014996	5.702E-07	1.500E-04	6	p21, Frizzled, Shc, MM	1.520E-01	2.544E-01	1	Frizzled
3	Development_NOTCH-induced EMT	19	4.095E-05	0.00146043	1.241E-01	3.707E-01	1	NOTCH4	4.095E-05	1.460E-03	3	NOTCH1 (NICD), NOTCH1 (NEXT), NOTCH1 receptor
4	Development_Regulation of epithelial-to-mesenchymal transition (EMT)	64	7.567E-05	0.00767064	7.567E-05	7.671E-03	5	NOTCH4, Frizzled, MMP	2.256E-02	2.195E-01	2	Frizzled, NOTCH1 receptor
5	Cytoskeleton remodeling_TGF, WNT and cytoskeletal remodeling	111	1.113E-04	0.00767064	1.113E-04	7.671E-03	6	MRLC, p21, Frizzled, S1	7.472E-03	1.148E-01	3	MELC, Frizzled, Tcf(Lef)
6	Development_MAG-dependent inhibition of neurite outgrowth	37	1.167E-04	0.00767064	1.167E-04	7.671E-03	4	MRLC, NGFR (ICD), NG	1.267E-01	2.544E-01	1	MELC
7	Translation_Non-genomic (rapid) action of Androgen Receptor	40	2.617E-03	0.07647765	2.617E-03	7.648E-02	3	Frizzled, Shc, WNT	1.363E-01	2.544E-01	1	Frizzled
8	Development_Melanocyte development and pigmentation	49	4.670E-03	0.08058998	4.670E-03	8.059E-02	3	p90RSK1, Frizzled, WN	1.644E-01	2.544E-01	1	Frizzled
9	Cytoskeleton remodeling_Cytoskeleton remodeling	102	5.371E-03	0.08058998	5.371E-03	8.059E-02	4	MRLC, p21, Shc, Zyxin	5.295E-02	2.544E-01	2	MELC, Tcf(Lef)
10	Development_WNT signaling pathway. Part 2	53	5.822E-03	0.08058998	5.822E-03	8.059E-02	3	Frizzled, WNT, Cyclin D	1.581E-02	1.692E-01	2	Frizzled, Tcf(Lef)

-2, WNT, Tropomyosin-1

hc, WNT, Cyclin D1

FR(TNFRSF16), NGFR (CTF)

T3A

Biology Open • Supplementary information

Table S5. List of primers sequences

Primer name	Sequences (5' to 3')
mGreb1 For	GCCACGGGGCGTCCGGCCCTTTC
mGreb1 Rev	ACCGCGCTGTGCAGGCGGGGGA
chGreb1-For	ATCCGCAAGGGGAGTCTTTACC-3
chGreb1-Rev	GGTGAGGAGGATGAGGAGGTGA
zGreb1-For	AAGGAGCCACCCCTCTGCACATTCT
zGreb1-Rev	TTAGACGAAACCGCATTCGTCCTC
M1-RT-PCR-for	GGAGTCTGACCGCCAGTGACCAG
M1-RT-PCR-rev	AAGTGCATTACGTCCACATTCATCG
M2-RT-PCR-for	GCTTGTCTCTGAAGGAGGCTGAGCA
M2-RT-PCR-rev	ATTCTCCCTGTGGATCCATGCCAGT
